# Statement of retraction: CircRNA protein tyrosine phosphatase receptor type a suppresses proliferation and induces apoptosis of lung adenocarcinoma cells via regulation of microRNA-582-3p

**DOI:** 10.1080/21655979.2026.2730803

**Published:** 2026-10-01

We, the Editors and Publisher of the journal *Bioengineered*, have retracted the following article from publication.

Jiang, J., Ge, H., Yang, J., Qiao, Y., Xu, X., & Geng, Y. (2022). CircRNA protein tyrosine phosphatase receptor type a suppresses proliferation and induces apoptosis of lung adenocarcinoma cells via regulation of microRNA-582-3p. *Bioengineered*, 13(5), 12182–12192. https://doi.org/10.1080/21655979.2022.2073319

Since publication, significant concerns have been raised by a third party about the integrity of the data and reported results in the article, including concerns about alleged image duplication with similar images from unrelated articles. Further assessments by the Taylor and Francis Publishing Ethics and Integrity team found further concerns with the article.

When approached for an explanation, the authors did not respond to our queries and so these serious concerns remain unaddressed.

As verifying the validity of published work is core to the integrity of the scholarly record, we are therefore retracting the article. The authors have been informed of this decision.

We have been informed in our decision-making by our policy on publishing ethics and integrity and COPE guidelines. 

The retracted article will remain online to maintain the scholarly record and will be digitally watermarked on each page as ‘Retracted’. 

